# Inoculum growth impacts *Salmonella* and Shiga-toxin producing *Escherichia coli* resilience on wheat grain

**DOI:** 10.1128/aem.00177-25

**Published:** 2025-03-31

**Authors:** Yawei Lin, Carolyn Peterson, Bradley P. Marks, Teresa M. Bergholz

**Affiliations:** 1 Department of Food Science and Human Nutrition, Michigan State University 437639 , East Lansing, Michigan, USA; 2 Department of Biosystems and Agricultural Engineering, Michigan State University 172887 , East Lansing, Michigan, USA; INRS Armand-Frappier Sante Biotechnologie Research Centre, Laval, Quebec, Canada

**Keywords:** desiccation tolerance, tempering, adaptation, low moisture food

## Abstract

Previous studies have shown that using a surface growth method for preparing inoculum impacted pathogen population stability and inactivation kinetics. Here, we quantified the desiccation survival and responses to tempering treatment of *Salmonella* and Shiga-toxin producing *Escherichia coli* (STEC) on wheat grain when grown using five different methods: broth, lawn-aerobic, lawn-anaerobic, lawn-acid-adapted, and lawn-low inoculum. Three strains of *Salmonella* and STEC each were individually inoculated onto wheat grain, conditioned to 0.45 *a*_w_ and stored up to 24 weeks. Pathogen survival curves on grain were different when inoculated with a lawn-grown culture compared to broth-grown. Acid adaptation of STEC led to increased (*P* < 0.001) tolerance to desiccation, while no change in *Salmonella* desiccation tolerance was observed. While most survival curves were better described by the log-linear model, survival of pathogens from broth-grown inoculum was better characterized by the Weibull model. Five-strain cocktails of each species were inoculated onto wheat, stored for 1, 2, 7, 28, and 84 days, followed by tempering for 18 h with three chemical interventions: water (control), 800 ppm chlorine, 5% lactic acid (LA) + 26.6% NaCl and 5% sodium bisulfate (SBS). These treatments led to different (*P* < 0.001) average reductions of 0.52, 1.04, 1.43, and 1.96 log CFU/g, respectively. The length of storage and inoculum growth method affected (*P* < 0.001) pathogen survival during tempering. In general, pathogens inoculated on grain survived better during tempering when inoculum was prepared by acid-adapted ≥ lawn-aerobic ≥ low inoculum > lawn-anaerobic = broth.

**IMPORTANCE:**

Outbreaks linked to wheat flour increased interest in evaluating pathogen survival kinetics. With minimal information on how foodborne pathogens contaminate wheat grain, the “worst-case scenario” should be identified to characterize pathogen survival kinetics on grain and be used to assess the effectiveness of food safety interventions. Using an antimicrobial solution during wheat tempering, an existing unit operation where grain is exposed to water prior to milling into flour can be a cost-effective way to mitigate the risk of foodborne pathogens. The lack of consistent inoculum preparation methods makes it difficult to compare results across studies evaluating tempering treatments. We assessed five different inoculum growth methods to quantify pathogen survival during desiccation and long-term storage and pathogen inactivation efficacy of several existing tempering solutions. In addition, these data provide insights on statistically important parameters to consider for low-moisture food challenge study experimental design, such as inoculum growth, inoculation level, and pathogen adaptation.

## INTRODUCTION

The incidence of foodborne outbreaks linked to wheat flour (1–3) in recent years has led to an interest in understanding pathogen resilience in flour and its raw material, wheat grain. Wheat grain is a low-moisture food (LMF) with water activity (a_w_) of around 0.45–0.55, which does not support bacterial growth. The main foodborne pathogens of interest include *Salmonella* and Shiga-toxin producing *Escherichia coli* (STEC); whereas *Salmonella* is more prevalent in recalls and outbreaks linked to LMFs in general, STEC is more commonly associated with flour-related outbreaks (4). Several studies have shown that these pathogens can survive on wheat grain and in flour for an extended period (5–8).

According to the National Advisory Committee on Microbiological Criteria for Foods, the inoculum growth method used for challenge studies should simulate how bacteria are grown or introduced to a food matrix in nature (9). Experimental design elements such as inoculum preparation could lead to different results and, therefore, interpretations. However, due to the knowledge gap of how pathogens are likely to be introduced to LMFs in nature, assessing multiple pre-growth conditions may help us better capture the survival patterns of pathogens after contamination (10). It is also reasonable to address the “worst-case scenario” of bacterial adaptation to adverse environments. Identifying inoculum growth methods that increase pathogen resistance to relevant stresses in the examined food system would be necessary to meet this recommendation.

Utilizing contaminated flour from an outbreak in Canada, researchers determined that the STEC O121 strain associated with the outbreak was able to persist in flour at levels associated with infections for up to 2 years (6). In laboratory studies with inoculated flour, pathogen survival has been found to vary, declining in some cases from 8 log CFU/g to the limit of detection (2 log CFU/g) within 16 weeks for *Salmonella* and STEC (11, 12), significantly faster than the observations from Michael et al. (7), who reported 1.4–2.4 log CFU/g reductions for *Salmonella*, and 2.8–5.0 log CFU/g reductions for STEC O121 after 12–25 weeks of storage. One of the reasons for these observed differences could be how the inoculum was grown, in which the pathogens were prepared in broth for the previous study, and lawn-grown inoculum was employed for the latter case. Moreover, studies have shown significantly greater survival of *Salmonella* when grown on an agar surface (lawn growth) compared to when grown in a broth prior to inoculation of various LMFs, such as almonds (13, 14), cocoa butter oil, crushed cocoa, hazelnut shells, cocoa beans (14), peanut butter (15), soy flour, nonfat dry milk, and all-purpose flour (16). Additionally, a meta-analysis applying linear regression to identify important factors that influence pathogen survival in LMFs found that inoculum preparation, broth vs lawn growth, had a significant impact on *Salmonella, E. coli*, and *Listeria monocytogenes* survival (17). In addition to the impact on desiccation survival, previous studies have also shown that inoculum growth method impacts pathogen thermal tolerance on LMFs (15, 18). However, whether the inoculum growth method could impact pathogen tolerance to chemical treatments when applied to LMFs remains unknown.

Tempering is an essential step in flour milling, where moisture is added to wheat grain to allow easier separation of the bran layer from the endosperm. Given that wheat is a low-cost commodity, tempering using solutions with a sanitizer effect is relatively cost-effective for the milling industry to reduce potential contamination of flour, and compared to various thermal treatments, may be less likely to compromise flour quality (19). When water is used as a tempering solution, the bacterial population on wheat grain did not significantly change over time (20, 21), while the use of chlorinated water at 800 ppm (22), 5% sodium bisulfite (SBS) solution (23), and 5% lactic acid (LA) + 26.6% sodium chloride (NaCl) (24) as chemical tempering solutions have led to different levels of bacterial inactivation. These chemical treatments pose acid, osmotic, and/or oxidative stresses, either individually or in combination. The impact of inoculum growth method on bacterial tolerance to these stresses, after desiccation on the surface of a LMF, has yet to be explored.

Most survival studies on LMFs have been conducted with different serovars of *Salmonella*, while the impact of inoculum growth method on STEC behavior on LMFs has been studied less frequently. Abiotic surfaces, such as filter discs, have been used to characterize pathogen adaptation to desiccation, which led to other findings regarding inoculum growth methods; Suehr et al. found that acid-adapted STEC O121 cells demonstrated improved desiccation survival over 30 days of storage, which was not observed for *Salmonella* or STEC O157:H7 (25). Additionally, it has been suggested that oxygenation during growth may alter the metabolism and physiology of pathogens during desiccation (26). Streufert et al. found that *Salmonella*, STEC, and *Listeria monocytogenes* had greater desiccation survival on filter discs when the inoculum was grown under aerobic conditions on agar media, compared to inoculum grown in liquid media or under anaerobic conditions on agar media (27). A higher inoculum level also provides pathogens with a protective effect during desiccation survival (18, 27), which was previously observed for *Salmonella* survival on filter discs but not for STEC (28).

Therefore, the objective of this study was to quantify long-term survival of *Salmonella* and STEC on wheat grain, using modeling as a tool to assess the impact of inoculum growth method on pathogen long-term survival. We also assessed the impact of inoculum growth method for *Salmonella* and STEC cocktails inoculated onto wheat grain for their response to various chemical tempering treatments. These data provide insights into parameters to consider when designing experiments to evaluate tempering treatments and also provide a direct comparison of some of the currently proposed tempering treatments of interest to the wheat milling industry.

## RESULTS AND DISCUSSION

### Pathogen concentration loss during initial adaptation to low *a*_w_ on wheat grain

**Fig 1:**
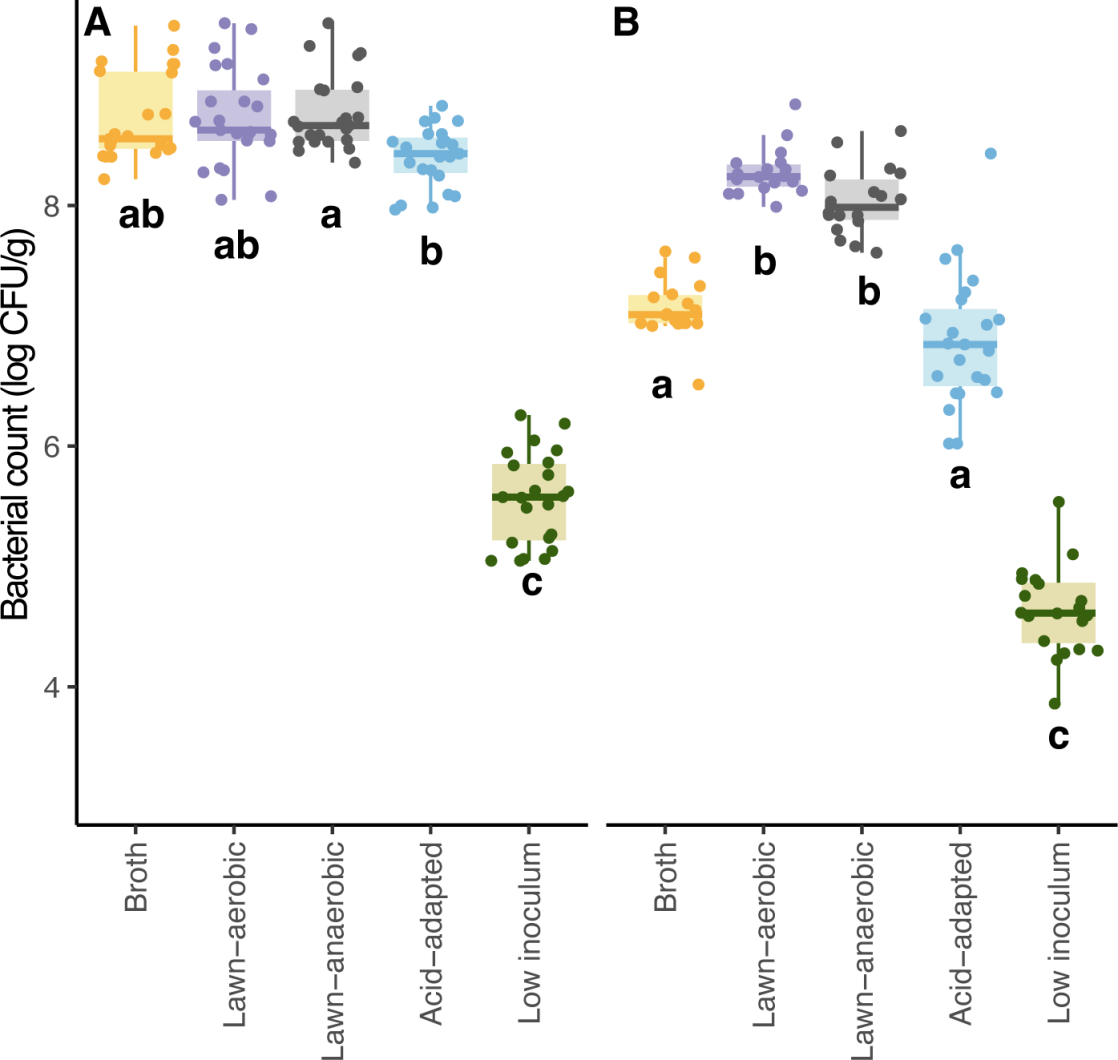
Bacterial population levels of (**A**) inoculum and (**B**) after water activity equilibration on wheat grain across inoculum growth methods for *Salmonella* and STEC. Different letters within each panel indicate mean values are significantly different (*P* < 0.05) and were results of three replicates (*n* = 3).

Excluding the pathogens prepared with a low inoculum, which has a lower target inoculation level of ~4 log CFU/g, other inoculum conditions started at similar (*P* = 0.437) levels of ~8.5 log CFU/g (Fig. 1A). Within each inoculum growth method, resulting cell densities did not differ significantly (*P* = 0.598) among strains (Table S1), so further comparisons are made with the STEC and *Salmonella* data presented in aggregate.

**Fig 2:**
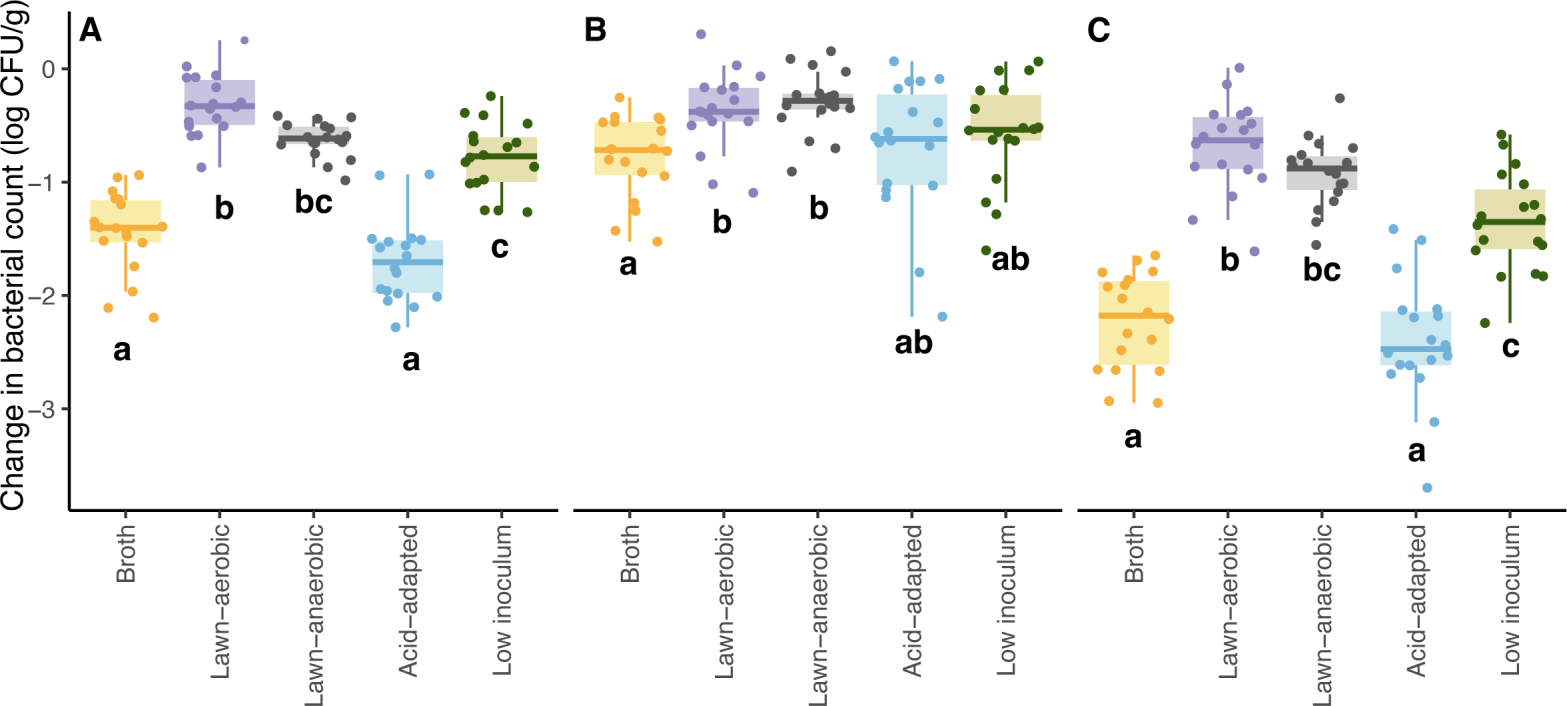
Changes in bacterial population (**A**) from inoculum to wheat grain, (**B**) during water activity equilibration, and (**C**) from inoculum to after water activity equilibration, for *Salmonella* and STEC. Different values within each panel indicate mean values are significantly different (*P* < 0.05) and were results of three replicates (*n* = 3).

When inocula were applied to grain, significantly greater (*P* < 0.001) changes in bacterial densities were found for pathogens that were grown in broth and grown under acid-adapted conditions (Fig. 2A), with average changes of −1.4 ± 0.4 and −1.7 ± 0.4 log CFU/g, respectively, compared to those grown with the other three methods, with average changes of −0.3 ± 0.3 (lawn-aerobic), −0.6 ± 0.2 (lawn-anaerobic), and −0.8 ± 0.3 log CFU/g (low inoculum). Average changes in pathogen levels on grain during *a*_w_ equilibration (Fig. 2B) were −0.8 ± 0.4 (broth), −0.4 ± 0.3 (lawn-aerobic), −0.3 ± 0.3 (lawn-anaerobic), −0.7 ± 0.6 (acid-adapted), −0.6 ± 0.5 (low inoculum) log CFU/g. Even though the inoculum prepared by lawn-aerobic and low-inoculum methods grew similarly, pathogen reduction on grain was significantly different (*P* < 0.001) during initial adaptation (Fig. 2C) for those with a low inoculum compared to the lawn-aerobic grown inoculum.

Initial adaptation to the low-moisture food environment led to the greatest pathogen reduction (Fig. 2), prior to *a*_w_ equilibration. Even though applying inoculum to wheat significantly increased the *a*_w_ from 0.45 to about 0.7–0.8, it was still below the *a*_w_ necessary for bacterial multiplication. Previous research (20) found average reductions of 0.75 and 0.97 log CFU/g for *Salmonella* and STEC cocktails prepared by lawn-inoculum on wheat grain after 48 h of drying, with initial pathogen levels on wheat ~4 log CFU/g, which was similar to what we found for low inoculum, with a 0.57 log CFU/g reduction during *a*_w_ equilibration.

During *a*_w_ equilibration, where *a*_w_ of the inoculated wheat gradually decreased to the pre-inoculation level (*a*_w_ = 0.45), pathogen reduction on grain was significantly greater for those prepared by broth and the acid-adapted inoculum (Fig. 2B). The 48 h equilibration period (Fig. 2B) led to lower pathogen loss compared to the initial adaptation during inoculation for all inoculum growth methods except for the lawn-aerobic inoculum method, which had a similar reduction between these two time points examined.

In summary, the levels of inoculated pathogens on grain were significantly different after *a*_w_ equilibration (Fig. 1B). Except for the low inoculum, the average starting population (Fig. 1) was not significantly different between lawn-aerobic and lawn-anaerobic grown inoculums and was not significantly different between broth and acid-adapted inoculum, but the difference was significant between these two groups. The average starting levels of pathogens on wheat grain after *a*_w_ equilibration were 6.4 ± 0.3 (broth), 7.9 ± 0.3 (lawn-aerobic), 7.7 ± 0.3 (lawn-anaerobic), 6.0 ± 0.4 (acid-adapted), and 4.1 ± 0.4 (low inoculum) log CFU/g. Due to the significant (*P* < 0.05) changes in pathogen population among inoculum growth methods during this stage, and to exclude changes in *a*_w_ as a confounder, only the pathogen count data after *a*_w_ equilibration were included for modeling of survival parameters.

### Modeling long-term pathogen survival on wheat grain during storage

The *a*_w_ of the wheat grain throughout storage remained stable between 0.45 and 0.55 (Fig. S1). Wheat was stored in a controlled environment to minimize the effect of confounding parameters that could potentially impact the survival of pathogens on wheat. Survival parameters were estimated using the log-linear and Weibull models (Table 1). In most cases, the log-linear model and Weibull-Mafart model yielded similar values for the *D*-value and the first decimal reduction (*δ*), respectively, since the shape parameter was close to 1 (Table 1). By observing the data set and the fitted predictive curves (Fig. S2), we could clearly see a tailing effect in bacterial levels that contributes to shape parameters significantly (*P* < 0.001) lower than 1 on wheat grain inoculated with broth-grown cultures (mean = 0.60) and for wheat grain inoculated with *S*. Typhimurium (mean = 0.64).

**TABLE 1:** Parameters estimated by Weibull-Mafart’s and Log-linear model on pathogen survival on wheat grain over 168 days of storage (20°C, 60% relative humidity), of different inoculum growth methods

Strain	Inoculum	Weibull-Mafart’s model				Log-linear model		
		δ^*a*^ (day)	*P* ^*b*^	RMSE^*c*^ (log CFU/g)	AICc^*d*^	D-value^*e*^ (day)	RMSE^*c*^ (log CFU/g)	AICc^*d*^
*Salmonella* Enteritidis PT30	Broth	17.8 ± 4.1	0.5 ± 0.1	0.33	−133.7	56.0 ± 6.8	0.42	−90.5
	Lawn-aerobic	82.1 ± 7.4	1.1 ± 0.1	0.18	−178.1	79.0 ± 3.2	0.18	−180.1
	Lawn-anaerobic	75.7 ± 12.9	0.8 ± 0.1	0.18	−177.6	88.8 ± 6.3	0.19	−176.5
	Acid-adapted	37.1 ± 7.9	0.9 ± 0.1	0.41	−86.6	51.7 ± 2.6	0.42	−88.0
	Low inoculum	75.3 ± 19.3	1.2 ± 0.3	0.38	−86.3	68.4 ± 12.2	0.38	−88.0
*Salmonella* Typhimurium	Broth	18.5 ± 14.0	0.5 ± 0.2	0.36	−103.4	57.2 ± 5.0	0.45	−80.7
	Lawn-aerobic	27.4 ± 9.5	0.7 ± 0.1	0.42	−87.8	53.1 ± 7.2	0.46	−80.1
	Lawn-anaerobic	37.3 ± 10.7	0.7 ± 0.1	0.33	−113.6	55.4 ± 10.0	0.35	−109.3
	Acid-adapted	32.6 ± 5.1	0.6 ± 0.1	0.57	−52.6	64.8 ± 19.7	0.61	−47.7
	Low inoculum	29.6 ± 16.2	0.6 ± 0.1	0.21	−105.4	50.6 ± 10.8	0.26	−91.5
*Salmonella* Mbandaka	Broth	7.3 ± 3.2	0.6 ± 0.1	0.41	−69.8	25.5 ± 2.4	0.55	−46.3
	Lawn-aerobic	32.2 ± 4.6	0.9 ± 0.0	0.39	−96.7	36.0 ± 5.5	0.39	−98.4
	Lawn-anaerobic	46.1 ± 7.7	1.3 ± 0.35	0.51	−67.4	31.4 ± 6.7	0.55	−60.9
	Acid-adapted	36.4 ± 9.8	1.2 ± 0.2	0.42	−81.0	29.8 ± 3.7	0.42	−83.1
	Low inoculum	21.7 ± 5.2	0.7 ± 0.0	0.29	−89.0	35.5 ± 4.0	0.32	−82.7
STEC O157:NM	Broth	14.7 ± 7.1	0.7 ± 0.1	0.27	−94.3	24.2 ± 4.0	0.33	−78.7
	Lawn-aerobic	38.4 ± 7.6	1.0 ± 0.1	0.42	−88.0	40.1 ± 2.6	0.42	−90.2
	Lawn-anaerobic	36.5 ± 7.9	0.9 ± 0.1	0.32	−117.5	45.4 ± 3.6	0.33	−116.7
	Acid-adapted	81.6 ± 43.1	1.1 ± 0.7	0.42	−78.3	91.9 ± 26.4	0.45	−80.6
	Low inoculum	28.8 ± 6.4	1.0 ± 0.1	0.23	−98.5	32.6 ± 1.1	0.23	−99.8
STEC O26:H11	Broth	11.9 ± 3.4	0.7 ± 0.0	0.34	−81.2	26.2 ± 4.0	0.44	−60.8
	Lawn-aerobic	41.3 ± 13.2	1.0 ± 0.2	0.37	−102.2	43.5 ± 5.2	0.37	−103.9
	Lawn-anaerobic	40.8 ± 13.7	1.0 ± 0.3	0.55	−59.6	42.3 ± 10.6	0.54	−61.9
	Acid-adapted	69.4 ± 48.6	1.4 ± 1.2	0.56	−57.7	70.7 ± 8.1	0.55	−60.0
	Low inoculum	21.6 ± 7.6	0.7 ± 0.2	0.25	−94.8	35.6 ± 0.9	0.29	−85.3
STEC O121:H19	Broth	12.7 ± 10.1	0.7 ± 0.3	0.36	−73.9	23.1 ± 2.3	0.42	−63.5
	Lawn-aerobic	26.9 ± 5.4	0.8 ± 0.0	0.42	−87.8	36.4 ± 3.1	0.43	−85.8
	Lawn-anaerobic	27.2 ± 6.2	0.8 ± 0.2	0.50	−68.6	43.2 ± 9.6	0.52	−66.9
	Acid-adapted	76.8 ± 18.2	1.0 ± 0.3	0.48	−98.6	83.1 ± 5.6	0.38	−100.8
	Low inoculum	33.1 ± 13.7	1.0 ± 0.2	0.42	−49.0	33.0 ± 10.2	0.42	−50.4

^*a*^ Mean ± standard deviation of first decimal reduction estimated by Weibull-Mafart’s model (*n* = 3).

^*b*^ Mean ± standard deviation of shape parameters estimated by Weibull-Mafart’s model (*n* = 3).

^*c*^ RMSE of the model combining all three biological replicates.

^*d*^ AICc values of the model combining all three biological replicates.

^*e*^ Mean ± standard deviation of *D*-values estimated by Log-linear model (*n* = 3).

In summary, the model selection depended on both inoculum growth method and strain. While the inactivation pattern of pathogens on grain inoculated with a broth-grown culture and for *S*. Typhimurium grown using all the methods was better characterized by the Weibull-Mafart model with a clear tailing effect, in most other cases, a linear model will be appropriate for pathogen inactivation estimation.

### Broth inoculum: is it less desiccation tolerant compared to other agar-grown inoculums?

For the Weibull-Mafart model, pathogens on grain inoculated with broth-grown cultures had shape parameters lower than 1 (*P* < 0.001) indicating a tailing effect. A strong tailing effect was also observed for 7 serovars of STEC and *Salmonella* with broth-based inoculum (11, 12) showing that inoculum prepared by the broth-grown method may need a longer time to adapt to a low water activity environment. For STEC serovars O26, O103, O111, and O157, the shape parameters ranged from 0.46 to 0.70. In addition, significant reductions of pathogen numbers on LMFs during the early stages of storage have been shown when using a broth-grown inoculum, and the rate of decline decreases after a period of time (13–15, 29). Some studies suggested 5–7 days of equilibration at controlled *a*_w_ before experimentation may result in greater repeatability and stability of pathogens, as evaluated in LMF thermal studies (30). Therefore, using different modeling approaches could better characterize the survival of pathogens on LMFs, especially under cases such as those inoculated with broth-grown cultures, where the reduction happens more rapidly during initial adaptation.

Simply presenting the overall reduction does not communicate the whole picture of pathogen dynamics during long-term survival. For example, earlier studies evaluated the impact of broth-grown vs lawn-grown inocula on pathogen survival on LMFs by assessing overall pathogen reduction rather than modeling survival parameters. For example, on almonds, the levels of inoculated *S*. Enteritis PT30 after 24 h of drying dropped significantly from 8.1 to 4.4 log CFU/g for the broth-grown inoculum compared to a smaller decline from 9.7 to 8.0 log CFU/g for the lawn-grown inoculum (13). During subsequent storage, the *Salmonella* levels dropped below 1 log CFU/g after 224 days for the broth-grown inoculum, compared to the lawn-grown inoculum, which remained at >3 log CFU/g after 550 days. However, the author also indicated that during the subsequent storage, the rate of *S*. Enteritidis PT30 reduction was not significantly different between broth- and lawn-grown inoculum. Similar results were found for *S*. Tennessee and Oranienburg in peanut butter (15).

Additionally, broth-grown inoculum led to greater pathogen reduction than lawn-grown inoculum for multiple *Salmonella* strains in cocoa butter oil, crushed cocoa and hazelnut shells, and cocoa beans (14). The same study also found no significant difference in the survival of *S*. Enteritidis PT30 between lawn vs broth inoculum when inoculated onto almonds. This finding contradicted what was observed in cocoa products; the author hypothesized this may be associated with antimicrobial properties of cocoa, but this hypothesis did not hold for the other *Salmonella* serovar used in the study, *S*. Oranienburg. These findings suggest that pathogens grown by broth inoculum potentially require more time to adapt to low moisture environments and could be food matrix and bacterial isolate dependent.

The difference in survival patterns between broth-grown inoculum and other agar-based methods may be due to the difference in physiological state of the bacterial cells. In some previous studies, broth- and lawn-grown inocula were also referred to as planktonic and sessile cells (15, 27, 28), which is defined by whether the bacteria are freely existing in solution or attached to a surface (31). Sessile cells can form biofilms, which exhibit distinct changes in physiological characteristics compared to planktonic cells, including the production of extracellular polymeric substances, changes in growth rate, gene expression, and increased resistance to sanitizers and antibiotics (32, 33). Liu et al. (29) also showed that more long-term desiccation-related genes were upregulated when *S*. Enteritidis was grown as a lawn compared to in broth. Sessile cells, such as those grown on agar surfaces, may be able to adapt to desiccation conditions at an earlier stage compared to planktonic cells.

### Impact of strain and inoculum growth methods on pathogen long-term survival on wheat grain

**Fig 3:**
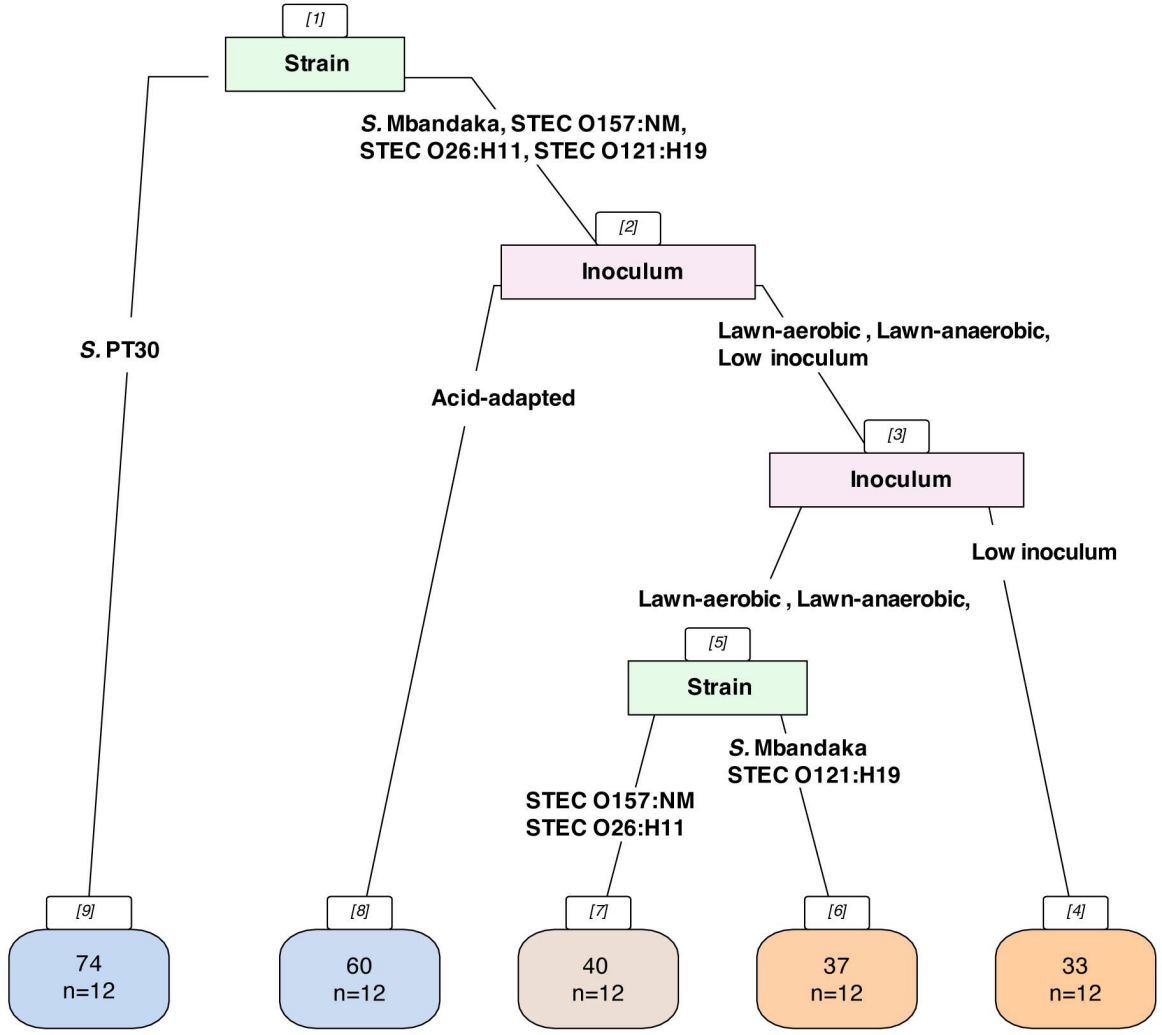
Classification decision tree for the log-linear model parameters for pathogen survival during storage of inoculated wheat. The boxes in colors indicate the parameter that led to optimal variance reduction for the model, with branches that represent the condition within the variables, leaves in the bottom include the average *D*-values in days, and *n* equals the number of data that fell into the classification. The number inside the bracket represents the nodes of the tree.

The classification tree analysis revealed differences in *D*-values for pathogen survival on grain, depending on inoculum growth methods and strains. *S*. Enteritidis PT30 had higher (*P* < 0.001) *D*-values on wheat grain compared to the other strains, under all the agar-based inoculum growth methods (Fig. 3, Node 9). We evaluated curli production using the Congo Red assay and found that only *S*. Enteritidis PT 30 produced curli (Fig. S3). Curli production is known to be involved in biofilm formation (34), and previous studies indicate that colonies exhibiting the rdar (red, dry, and rough) morphology, such as *S*. Enteritidis PT 30 here, to be more tolerant to long-term desiccation on nutrient-depleted plastic surface, enhancing the resistance of *Salmonella* to desiccation (35, 36), which could be a contributing factor in the difference in *D*-values observed here. For the other strains, pathogens prepared from the acid-adapted inoculum had higher (*P* < 0.001) *D*-values (Fig. 3, Node 8), and those from the low-inoculum method had lower (*P* = 0.007) *D*-values (Fig. 3, Node 4), compared to the lawn-aerobic and lawn-anaerobic grown inoculum. Between these two inoculum growth methods, STEC O157:NM and STEC O26:H11 had higher (*P* = 0.04) *D*-values (Fig. 3, Node 7), compared to *S*. Mbandaka and STEC O121:H19 (Fig. 3, Node 6). The observations from broth-grown inoculum, and those from *S*. Typhimurium, where a significant (*P* < 0.001) tailing effect was observed, were excluded from this classification tree since the log-linear model was not able to characterize their survival pattern.

#### Lawn-aerobic

Lawn-aerobic grown inoculum was found to have less variability in *D*-values compared to other inoculum growth methods within the same strain. While *S*. PT30 had the largest *D*-value of 79 days, for other strains, the *D*-value of pathogens grown with the lawn-aerobic method ranged from 36 to 43 days. Lauer et al. reported that *D*-values for *Salmonella* on wheat grain ranged from 22.9 ± 2.2 to 25.2 ± 1.0 weeks (160–176 days/ log), and STEC had *D*-values from 11.3 ± 0.6 to 13.1 ± 1.8 weeks (79–92 days/ log) based on the log-linear model when a lawn-based inoculum was used (5). Michael et al. (7) studied the survival of *Salmonella* and STEC O121 cocktails on wheat flour inoculated with lawn-grown cultures and found the STEC population decreased from 7.6 ± 0.18 to 2.0 ± 0.40 log CFU/g after 360 days of storage (~64 days/log reduction), and the *Salmonella* population decreased from 7.8 ± 0.1 to 2.9 ± 0.6 log CFU/g (~73 days/log reduction). The results in the current study showed a smaller *D*-value for both *Salmonella* and STEC compared to the wheat grain study, but the *D*-value was similar for *Salmonella* when compared to the flour study. Both studies align with our finding that *Salmonella* species in general survive better on grain compared to STEC.

#### Lawn-aerobic vs anaerobic

Lawn cultures grown under anaerobic conditions did not lead to significantly (*P* = 0.10) different *D*-values on grain within each strain compared to the lawn-aerobic grown inoculum (Fig. 3, Node 5), showing that agar-based inoculum preparation methods for strains in an anaerobic environment did not impact desiccation tolerance. A previous study found that initial *Salmonella* and STEC desiccation tolerance after 24 h on filter disks for cells prepared by lawn-grown inoculum under aerobic conditions was greater than or equal to that when grown on agar media under anaerobic conditions (27). In our study, the reduction of the cells prepared by the lawn-anaerobic growth during initial adaptation compared to the cells grown by lawn-aerobic method (Fig. 2) was not significant (*P* = 0.97).

#### Higher inoculum level led to greater pathogen survival on wheat grain

The *D*-values for pathogens on grain with low-inoculum levels were lower than the *D*-values for pathogens with lawn-aerobic/anaerobic inoculum for all strains except *S*. PT30 (Fig. 3, Node 3). In pathogen inactivation studies, researchers typically use a high number of bacteria (~8 to 9 log CFU/g) for inoculation to ensure sufficient quantification of the reduction. In some cases, laboratory studies were designed based on regulatory guidelines from the government or industry (9), such as the mandatory 4-log reduction requirement for almonds grown in California (37). There is no regulatory guideline regarding the log-reduction required for treating wheat or flour, and we do not have much information on the route of contamination or the contamination level of wheat as a raw commodity, with only prevalence data indicating that the prevalence of pathogens in wheat was low, at 0.44% for STEC and 1.23% of *Salmonella* (38). In the absence of regulatory guidance, both inoculum levels could be used based on the research goal.

However, the impact of inoculum levels on pathogen survival during desiccation has been varied. For *Salmonella* on almond kernels, log reductions were similar for four different inoculum levels: 8, 5, 3, and 1 log CFU/g of almonds (13). A study with pecan kernels and raw peanuts also found that the inoculum levels of 6 or 3 log CFU/g did not have a significant impact on *Salmonella* survival (39). A desiccation study conducted on filter disks found that *Salmonella* and STEC population losses during an initial adaptation of 24 h increased as the inoculum level decreased (28). In our study, we also found the average reduction on grain during adaptation and *a*_w_ equilibration was significantly greater for pathogens with a low-inoculum level compared to those at a higher inoculum level even though they were grown using the same lawn-based method. Moreover, the study on filter disks also indicated that the persistence of pathogens after drying does not seem to be impacted by the initial cell level. However, in the current study, we found that the initial cell level still impacts pathogen persistence after initial adaptation for certain strains such as *S*. Mbandaka and all STEC strains used. Blessington et al. (40) study on in-shell walnuts suggested the rate of decline, especially at a lower inoculum level, had greater rate of decline during the first few weeks; this aligned with our findings for *S*. Typhimurium, Mbandaka, and STEC O26:H11, where the shape parameter of the Weibull model was lower than 1, indicating a tailing effect.

#### Acid-adapted inoculum impacts pathogen desiccation tolerance

Acidification of bacterial growth media can occur by the fermentation of glucose during growth. Lab media commonly used for inoculum growth in LMF research include LB, tryptic soy, and BHI broth/agar. While LB broth/agar and TSA do not contain glucose, TSB and BHI media contain a small amount of glucose. Minimal research has indicated whether the small amount (~2 to 2.5 g/L) in the medium leads to a difference in pathogen desiccation tolerance. By harvesting cells prepared by lawn inoculum from respective agar plates in 1 mL of 0.9% saline, Suehr et al. (25) found that the pH of cells collected from plates made with TSB and agarose had a slightly lower pH of 8.18–8.39 compared to TSA plate of 8.65–8.82. In the same study, the pH of cells on TSA plates with additional 1% glucose was significantly lower at 4.72 for *Salmonella*, and 5.28 for STEC. Though in our study, pH strips provide less accuracy compared to a pH meter, the pH value trends were still comparable with what we observed, where the pH of acid adapted inoculum was between 5 and 6, slightly lower than the pH of the broth inoculum and lawn-anaerobic inoculum at between 7 and 8, and the lawn-aerobic inoculum and the low inoculum at pH of around 7. Moreover, Suehr et al. observed less resistance of acid-adapted cells during drying, which we also observed (Fig. 2). Once the cells on the filter disks were dried, STEC O121 survival during subsequent ~30 days of storage was greater for acid-adapted cells than non-adapted, and the survival was not significantly different for *Salmonella* despite acid-adaptation. Both results were comparable to what was found in the current study, where acid-adapted STEC had higher (*P* < 0.001) *D*-values on wheat grain compared to other lawn-based inoculum growth methods (Fig. 3). On the other hand, the *D*-values for *Salmonella* from acid-adapted inocula were not different from non-adapted inocula (*P* = 0.72). However, we could not explain whether the distinct response to acid-adaptation for *Salmonella* and STEC was due to species differences or the selection of isolates. Future transcriptomic studies may help answer this question.

### Experimental design factors associated with pathogen inactivation efficacy during wheat tempering

**Fig 4:**
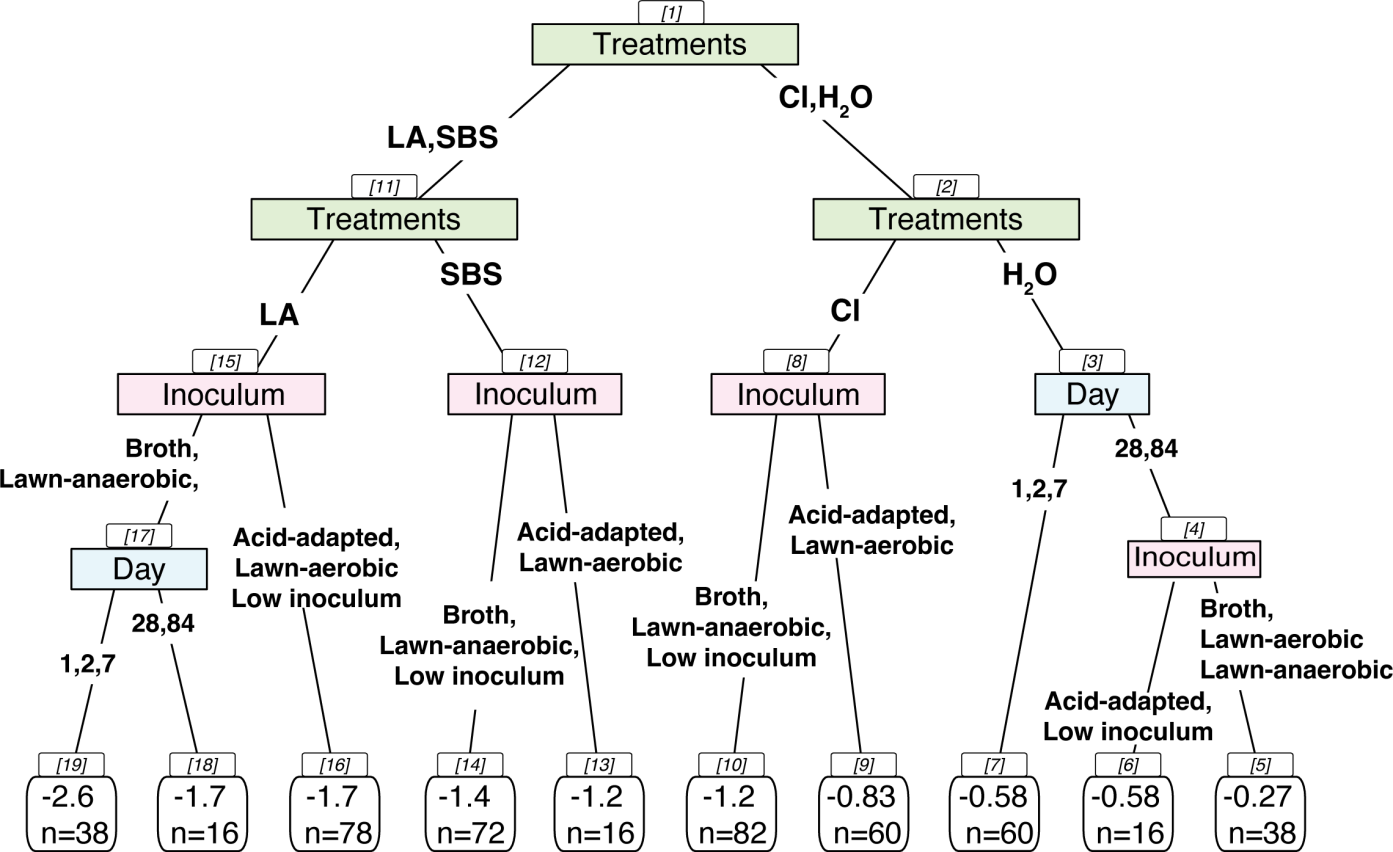
Classification decision tree for identification of statistically significant parameters for pathogen population changes during tempering. The boxes in colors indicate the parameter that led to optimal variance reduction for the model, with branches that represent the condition within the variables, leaves in the bottom include the average bacterial reduction of the classification in log CFU/g, and *n* equals number of observations within this classification. The number inside the bracket represents the nodes of the tree.

Factors that could potentially impact the inactivation efficacy of different tempering treatments were evaluated, including pathogen species (*Salmonella* vs STEC), inoculum growth method, and the number of days post-inoculation before tempering the grain, which allows various time for pathogens to adapt to a low moisture environment. The adaptation times utilized were 2, 7, 28, and 84 days post-inoculation. In addition, the samples were collected after 1 day of drying in a biosafety cabinet without *a*_w_ equilibration. The classification decision tree model identified variables that impact pathogen reduction during tempering based on ANOVA by optimally reducing the variance (Fig. 4). While the reductions of *Salmonella* and STEC were significantly different (*P* = 0.003) based on ANOVA, the variable did not appear in the final decision tree, indicating that it did not contribute significantly to the reduction of variance relative to other predictors under the tree-building criteria. This may be due to the complexity of data, as significant differences in bacterial reduction were observed between *Salmonella* and STEC when tempered with water (*P* = 0.012) and chlorinated water (*P* = 0.0012); no significant difference was observed when tempered with 5% SBS (*P* = 0.085) and 5% lactic acid + 26.6% NaCl (*P* = 0.123). All three tempering treatments, including the water control, were significantly (*P* < 0.001) different from each other (Fig. 4, Nodes 1, 2, 11).

For the water control, a longer adaptation time on grain prior to tempering led to significantly greater (*P* = 0.012) pathogen reduction on wheat (Fig. 4, Node 3). For inoculated wheat grain stored for 4 weeks or more, the broth, lawn-aerobic, and lawn-anaerobic grown inocula had lower (*P* = 0.002) reductions compared to pathogens prepared from acid-adapted inoculum and low inoculum. For wheat tempered with chlorinated water or 5% SBS (Fig. 4, Node 8, 12), pathogen reduction was higher (*P* < 0.001) on wheat inoculated with broth, lawn-anaerobic, and low inoculum compared to wheat inoculated with acid-adapted or anaerobic lawn-grown cultures. For wheat tempered with 5% lactic acid + 26.6% NaCl, the impact of adaptation time was significant (*P* = 0.012) only when the pathogens were prepared with broth and lawn-anaerobic inocula, leading to greater reduction (*P* = 0.002) than those prepared with lawn-aerobic, acid-adapted, or low-inoculum methods (Fig. 4, Node 15).

**Fig 5:**
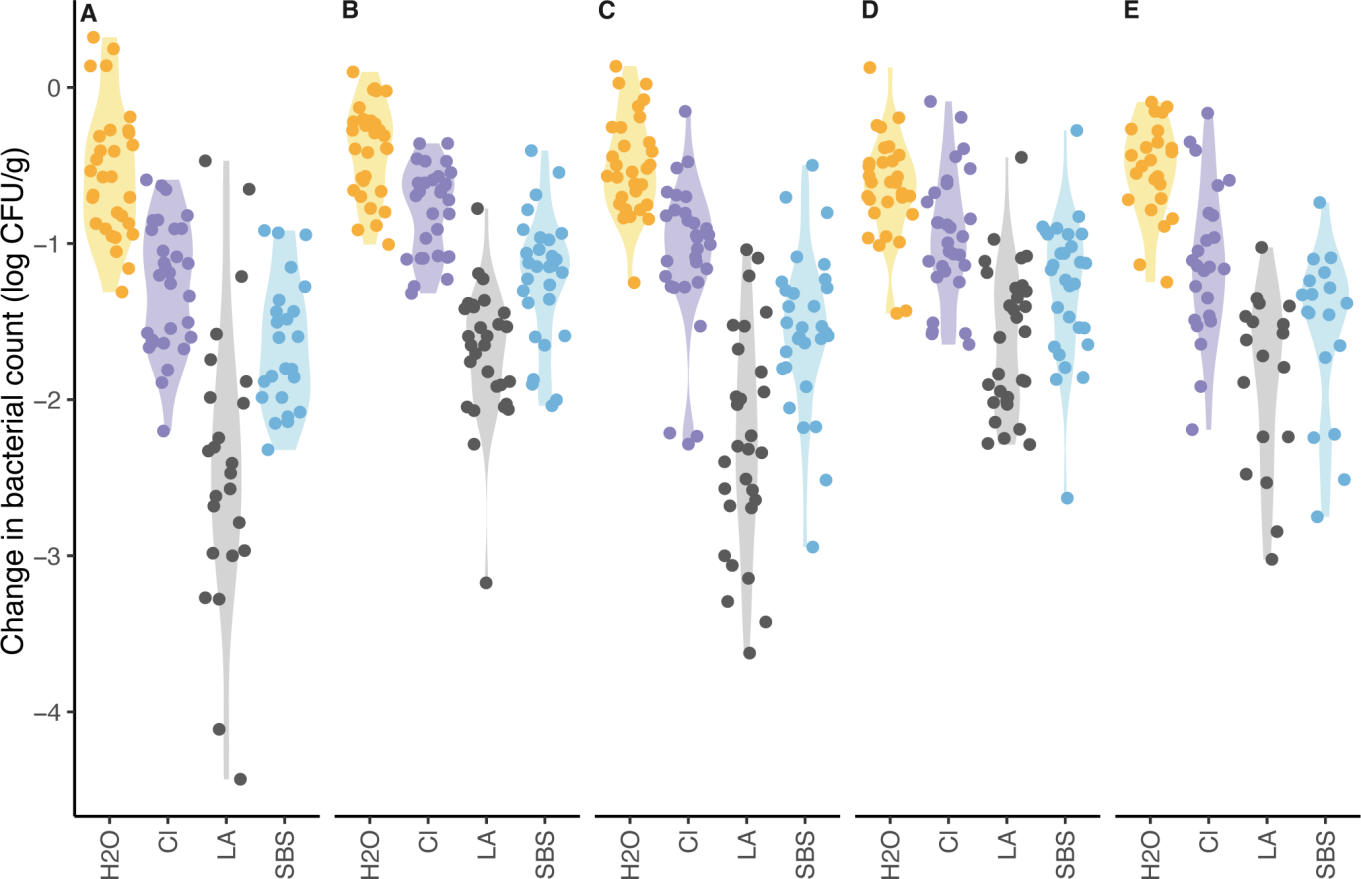
Changes in pathogen population levels on wheat grain for four tempering treatments by inoculum growth method. (**A**) broth, (**B**) lawn-aerobic, (**C**) lawn-anaerobic, (**D**) acid-adapted, and (**E**) low inoculum. Tempering treatments are listed on the *x*-axis and include H2O (water, negative control), Cl (800 ppm chlorine), LA (5% lactic acid + 26.6% NaCl), and SBS (5% sodium bisulfate).

The *a*_w_ of wheat grain, with *a*_w_ equilibration followed by subsequent storage, ranged from 0.45 to 0.50 for the samples used in the tempering study similar to numbers observed in the storage study. However, the *a*_w_ of wheat that was dried in the biosafety cabinet for 1 day, without equilibration, ranged from 0.37 to 0.65, which had large variability, even though no significant (*P* = 0.325) difference in pathogen reduction was observed compared to 2- and 7 days post-inoculation. The distributions of the bacterial population reduction, for different tempering treatments and inoculum (Fig. 5), showed the greatest variability for broth inoculum when grain was tempered with lactic acid. The average bacterial reduction with water across all inoculum growth methods was 0.5 ± 0.3 log CFU/g for *Salmonella* and 0.6 ± 0.3 log CFU/g for STEC. On average, the 5% lactic acid with 26.6% salt combination led to the greatest reduction for all inoculum growth methods (Fig. 4).

#### Inoculum growth method impacts efficacy of tempering treatments

The lawn-based and broth-based inocula also had an impact on bacterial survival during tempering. As this is the first study to examine the impact of inoculum growth method on wheat tempering efficacy, there is limited information for comparison. However, previous research examining the inoculum impact on pathogen thermal tolerance on LMFs found that growing the inoculum under lawn-aerobic conditions led to higher thermal resistance compared to broth-grown inoculum (15) and better laboratory reproducibility (18).

While the survival rates of pathogens prepared by the lawn-anaerobic method and lawn-aerobic method during long-term storage were similar, the reduction due to tempering treatments, except for the water control (*P* = 0.121), was higher for bacteria prepared by the lawn-anaerobic method compared to those prepared by the lawn-aerobic method. The availability of oxygen during growth could lead to alterations in the metabolism and physiology of pathogens, including resistance to various stresses (26). The mechanisms behind the phenotypic responses observed in the current study are not fully understood. Using a genetic tool such as transposon sequencing (41) may help identify genes that are essential for bacterial survival and may be impacted by oxygen availability.

#### Comparing tempering treatment efficacy

The standard tempering process, using water alone, does not lead to appreciable reductions in pathogen numbers. Similar to our findings, Jung and Harris found reductions of 0.43 and 0.45 log CFU/g for *Salmonella* and STEC cocktails when inoculated at ~4.0 log CFU/g on wheat grain (20). This reduction was not significantly different from the average reduction of 0.5 ± 0.3 log CFU/g we found for grain with low-inoculum levels (Table 2). Our previous study found that individual strains of *Salmonella* and STEC inoculated at ~6 log CFU/g had average reductions of 0.3 log CFU/g when tempering grain with water (22), which was similar to the reductions observed here when wheat was inoculated with lawn-aerobic grown inoculum. This aligned with the finding that a higher inoculum level may decrease the inactivation efficacy.

**TABLE 2:** Changes in pathogen population levels due to tempering

Treatment/inoculum	Broth	Lawn-aerobic	Lawn-anaerobic	Acid-adapted	Low inoculum
	Mean ± SD (log CFU/g)				
Chlorinated water (800 ppm)	−0.71 ± 0.35	−0.38 ± 0.21	−0.58 ± 0.38	−0.31 ± 0.30	−0.59 ± 0.36
5% LA + 26.6% Nacl	−1.78 ± 0.90	−1.29 ± 0.41	−1.77 ± 0.57	−0.98 ± 0.38	−1.33 ± 0.39
5% SBS	−1.01 ± 0.44	−0.82 ± 0.33	−1.06 ± 0.44	−0.63 ± 0.36	−1.20 ± 0.43

A previous study using 800 ppm chlorinated water to temper wheat grain led to a 2 log CFU/g reduction in *Salmonella* and 0.38 and 1.23 log CFU/g reduction in O157 and non-O157 STEC, respectively (22). The reduction of STEC found in the current study was within the range of what was found in the prior study. However, the reduction of *Salmonella* was lower in the current study; this may be due to differences in strain selection. Tempering with organic acid and saline was found to effectively reduce mesophilic bacteria (42); therefore, the study tested several organic acid and saline combinations for pathogen reduction and found that using a 5% LA + 26% NaCl solution was most effective, leading to 2.6 log and 2.4 log reductions for *Salmonella* and STEC, respectively, using a broth-based inoculum growth method. This finding was similar to what was found in the current study, with an average of −2.4 ± 0.9 log CFU/g changes when treating wheat inoculated with a broth inoculum using the acid-saline mixture. The use of lactic acid without salt was replicated by other studies, which led to 2 log reduction (broth inoculum, 5% LA) (23), and 1.40–2.38 log reduction (lawn-aerobic, 5.4% LA) (20), which were both within the range of what we found in the current study. Moreover, 5% SBS tempering led to 2 log reduction of a STEC O26 and O121 cocktail when prepared by broth inoculum (23), which was within the standard deviation of the reductions found in the current study. It was expected that the efficacy might be slightly different from previous studies that used other experimental design factors. For example, the difference between soft and hard wheat, the difference in initial moisture content of the sample acquired, will require different amounts of tempering solution to be added for treatment.

Most of the results we found were similar to previously reported research, based on each inoculum growth method, including broth and lawn-aerobic inoculum. Here, we provide a side-by-side comparison of the efficacy for each treatment when prepared using the same experimental design, where 5% LA + 26.6% NaCl led to the greatest pathogen reduction, followed by 5% SBS, and then 800 ppm chlorinated water. It is important to highlight again that broth inoculum led to a significantly higher reduction in pathogen population compared to lawn-aerobic inoculum for all three treatments we studied, reinforcing that choosing an appropriate experimental design is crucial for providing insight for commercial application.

The results in our study indicated that laboratory pre-adaptation to acid did lead to increased tolerance of pathogens to various tempering treatments, among which the LA and SBS solutions were both acidic at pH <2.0. While the desiccation tolerance of STEC was still higher than that of *Salmonella* cocktails in the tempering study, susceptibility to tempering treatments was not significantly different across species.

#### Other parameters that impact bacterial inactivation efficacy during tempering

The difference in bacterial reduction was mainly due to different tempering treatments and inoculum growth methods. In the current study, we also found that a longer adaptation time may lead to greater survivability of pathogens under some treatment conditions. This difference may be more significant for pathogens prepared by broth and lawn-anaerobic inoculum (Fig. 4). These are the two inoculum growth methods that involve less oxygenation.

Even though no significant difference (*P* > 0.05) was observed in the reduction of day 1 samples compared to days 2 and 7 across all treatments, the *a*_w_ of the day 1 sample dried in the biosafety cabinet varies with weather conditions. The *a*_w_ level impacts the thermal tolerance of pathogens, but no previous study has examined whether the *a*_w_ level would impact pathogen susceptibility to tempering treatments. Therefore, to control the experimental design, prior *a*_w_ equilibration and some drying or adaptation time should be recommended.

### Variation in survival among bacterial strains

*S*. Enteritidis PT30 had a distinct morphology on Congo Red (CR) Agar (Fig. S3) compared to other strains tested, implying its biofilm-forming ability (43). Even though the population loss of the strain was not significantly different from the other strains during initial adaptation, it had a significantly higher *D*-value during subsequent storage for the lawn-based aerobic/anaerobic inoculum and low-inoculum growth methods compared to the other *Salmonella* strains, including *S*. Typhimurium and *S*. Mbandaka. *S*. Typhimurium was also distinctly different from the other two *Salmonella* strains, as it also had a tailing effect when prepared by an inoculum growth method other than broth inoculum (Table 1), as the shape parameter ranged from 0.52 to 0.74 across all inoculum growth methods. Therefore, it is important to highlight that the selected strains could alter pathogen behavior in such challenge studies. The use of more resistant strains or a cocktail that could be representative of the species is critical for simulating the “worst-case scenario.” One limitation of this study is the use of rifampicin-resistant strains, where selection of the mutants may lead to additional genomic changes that could impact stress response. Through evaluation of next-generation sequencing of the isolates, we found that only *rpoB* was mutated between the parent and rifampicin-resistant mutant for all strains. However, further phenotypic evaluation is necessary to validate the use of rifampicin-mutant in such studies.

### Conclusion

In conclusion, our findings highlight that the inoculum growth method can significantly impact desiccation tolerance and subsequent survival of *Salmonella* and STEC on wheat grain. The reduction of pathogen population during initial adaptation was different from subsequent storage, and the survival pattern during long-term desiccation storage was not the same across inoculum growth methods and bacterial strains used, potentially depending on the biofilm-forming ability. In general, lawn-aerobic inocula led to more consistent experimental outcomes, and higher inoculum levels provided protection for pathogens during desiccation and subsequent chemical treatments. Acid adaptation of pathogens during growth significantly enhanced STEC tolerance to desiccation and chemical stress.

Moreover, this study selected a few previously reported tempering treatments, to assess their pathogen inactivation efficacy under uniform experimental design and found that initial adaptation potentially makes pathogens more resistant to the treatments. The results highlighted that it is critical to promote a standardized experimental design, especially for pathogen cultivation, making challenge studies more applicable in future discussions on flour safety interventions. Our research also raised questions about why and how inoculum cultivation could be so influential on a pathogen’s subsequent stress response, which will be investigated further using genomic tools.

## MATERIALS AND METHODS

### Wheat grain

A commercial blend of hard red spring wheat grain was kindly donated by Mennel Milling Company (Fostoria, Ohio). The grain was received in April 2022 and stored at 4°C in Whirl-Pak bags (Nasco Whirl-Pak, Madison, WI). Before inoculation, wheat was brought to room temperature, and the moisture content of uninoculated wheat was measured using a handheld moisture meter (MINIGAC1SG4, Dickey-John; Minneapolis, MN). The average moisture content of wheat was 12.3% ± 0.1%. The *a*_w_ of wheat was measured using the AQUALAB 4TE *a*_w_ meter (METER, Pullman, WA), and the average *a*_w_ was 0.45 ± 0.02. The water activity and moisture content of wheat grain were measured in duplicate for each of the three biological replicates.

### Bacterial strains

For the desiccation survival study, three strains of *Salmonella* and three strains of STEC were used: *Salmonella* Enteritidis PT30 (ATCC BAA-1045), Typhimurium (FSL S10-1766), and Mbandaka (698538), and STEC O157:NM (LJH1723), O26:H11 (LJH1728), and O121:H19 (PNUSAE002568). For the tempering experiment, two additional *Salmonella* strains, Tennessee (K4643) and Montevideo (488275), and two additional STEC strains, O157:H7 (SEA13B88) and O157:H7 (LJH1357), were added to make 5-strain cocktails of *Salmonella* and STEC, respectively. All the bacteria strains were selected for resistance to 80 µg/mL rifampicin previously (22), to distinguish them from the native microbiota on wheat.

### Inoculum growth methods and inoculation

Five inoculum growth methods of interest were used: (i) broth-based inoculum (Broth), (ii) lawn-based inoculum, aerobic (lawn-aerobic), (iii) lawn-based inoculum, anaerobic (lawn-anaerobic), (iv) lawn-based acid adapted inoculum, aerobic (acid-adapted), and (v) lawn-based low-density inoculum (low inoculum), aerobic. Briefly, the strains previously described were chosen based on their rifampicin resistance (80 µg/mL) and were preserved as freezer stocks. For each biological replicate, these strains were streaked onto Luria-Bertani (LB) agar (Invitrogen, Waltham, MA) with 80 µg/mL rifampicin (Sigma-Aldrich Corp., Milwaukee, WI) (LBr) and allowed to incubate at 37°C for 20 h. Subsequently, a single colony from each strain was isolated and introduced into 5 mL LBr broth and then incubated at 37°C for an additional 24 h. For the lawn-aerobic, lawn-anaerobic, and low-inoculum methods, 250 µL of the overnight culture was uniformly spread onto a LBr agar plate and incubated at 37°C for 24 h to achieve a confluent lawn growth, with the lawn-anaerobic samples being cultivated inside anaerobic GasPak jar (BD, Franklin Lakes, NJ) with anaerobic gas generator (Mitsubishi, Japan). For the acid-adapted inoculum, 250 µL of the overnight culture was spread evenly onto TSA agar (Neogen, Lansing, MI) with 1% additional glucose (Thermo Fisher Scientific, Waltham, MA) instead of LB and incubated at 37°C for 24 h .

For the broth inoculum, 250 µL of the overnight culture was introduced into a sterile centrifuge bottle containing 200 mL of LBr broth and incubated for 24 h. This broth inoculum was then harvested by centrifugation at 3,000 × *g* for 15 min. The resulting pellet was suspended in 2.5 mL of Butterfield’s buffer. For all lawn-based methods, the cells from the surface of the agar were carefully collected using a sterile L-shaped spreader, except for low inoculum, culture from five plates were pooled, and suspended in 2.5 mL of Butterfield’s buffer for inoculate 700 g wheat grain. For low inoculum, 0.1 mL was removed from lawn-aerobic inoculum, further diluted in 0.9 mL of Butterfield’s buffer, and ultimately 0.1 mL of this diluted culture was recombined with 2.5 mL of sterile Butterfield’s buffer for inoculation.

The bacterial concentration of the inoculum was quantified for each strain to examine the bacterial loss during initial adaptation to the wheat. The inoculum levels were calculated based on inoculum density (CFU/mL) and weight of wheat to determine the log CFU/g and compared with the pathogen levels on the wheat grain after inoculation.

For the tempering experiment, five strains from each species were prepared individually, and one plate from each serovar was pooled and suspended in 2.5 mL of Butterfield’s buffer to create the inoculum cocktail for all lawn-based methods. For the broth-based inoculum, five strains from each species were grown individually in 40 mL of LB broth; after 24 h of incubation at 37°C, the cultures were combined and harvested by centrifugation at 3,000 × *g* for 15 min. The resulting pellet was again suspended in 2.5 mL of Butterfield’s buffer for inoculation.

### Measurement of inoculum pH, biofilm-forming testing

The pH of the inoculum was tested in triplicate with pH test strip (MilliporeSigma, Burlington, MA) to determine if acidification occurred during bacterial growth. For the broth inoculum, the pH test strip was directly applied to supernatant after centrifugation by dipping, once the pellets were collected. For the agar-based inocula, after collecting the cells for inoculation, the pH test strip was applied to the surface of the solid medium using sterilized forceps.

The curli production of the bacteria was tested using LB without salt supplemented with 50 mg of Congo red (CR) (Sigma-Aldrich) per liter and 20 mg of brilliant blue (Sigma-Aldrich) per liter (34), with incubation at 30°C for 48 h. The results were interpreted by observation of the morphology described in reference 34.

### Inoculation, water activity equilibration, and storage

To inoculate the wheat for the storage study, inoculum was added directly to a Whirl-Pak bag containing 750 g wheat grain, and inoculum was massaged by hand to the wheat for 3 min. After mixing, kernels were tested for inoculation homogeneity by selecting three 11 g samples. By doing this, the inoculation level for day 0 was quantified; if the standard deviation of the samples was less than 0.5 log CFU/g, we assumed the wheat was inoculated homogenously. For the tempering experiment, two batches of 700 g of wheat were inoculated and mixed for each species per biological replicate. The inoculation consistency was assessed the same way as described above.

After inoculation, the wheat grain was transferred to a conditioning chamber (69 by 51 by 51 cm) for *a*_w_ equilibration for 48 h. The conditioning chamber includes custom control system to control chamber relative humidity within ~2% to 5% of target value (44) using desiccation column and hydration column filled with silica gel or water. Samples were placed in a commercially available aluminum foil tray (32 by 26 by 6 cm) for this period and mixed once after 24 h. Three samples were taken randomly to make sure *a*_w_ was equilibrated homogeneously across.

The equilibrated wheat was then transferred to a Mylar bag (22.5 by 28.9 cm; Uline, Pleasant Prairie, WI), sealed, and stored in a temperature-controlled fridge (Thermo Fisher Scientific) for further use. The relative humidity and temperature of the fridge were monitored using a HOBO Temperature/Relative Humidity 3.5% Data Logger (Onset, MA). The wheat grain for long-term desiccation survival was stored for 24 weeks after inoculation, with average temperature of 19.8 ± 0.3°C and relative humidity at 59.3% ± 3.5% (Fig. S1). The bacterial population was quantified within 2 h post-inoculation, after *a*_w_ equilibration, and on days 7, 14, 28, 56, 84, 112, 140, and 168.

### Inoculation and storage for pathogen inactivation during tempering

For the tempering experiment, 750 g of the inoculated wheat was dried in the biosafety cabinet at ambient temperature with the fan on for 24 h (day 1 sample), while the other wheat went through the *a*_w_ equilibration and storage as described above. The bacterial population was quantified on days 1, 2, 7, 28, and 84 after inoculation and tempering.

### Tempering solution preparation

Tempering solutions were prepared based on information in previous literature (22–24). The 800 ppm chlorinated water was prepared by dissolving calcium hypochlorite (Sigma-Aldrich) in sterile DI water, and the pH was adjusted to 6.5 by adding 25% (wt/vol) of citric acid (Sigma-Aldrich), and the concentration of total chlorine was verified using a test kit (CN-21P, Hach, Loveland, CO). The 5% lactic acid solution was prepared by diluting lactic acid of 85.0%–90.0% aq. (Thermo Fisher Scientific) with sterile DI water and mixing with 26.6% (wt/vol) of sodium chloride (Aldon Co., Avon, NY). The 5% sodium bisulfite (Sigma-Aldrich) solution was prepared by diluting with sterile DI water, and sterile DI water was used as a control.

### Tempering

For each biological replicate, a 50 g aliquot of inoculated wheat of the two bacterial species and four tempering treatments were placed into sterile 250 mL centrifuge bottles (Nalgene, Rochester, NY). Two inoculum growth methods were randomly selected for each experimental day, and a total of eight independent experiments were conducted for the three biological replicates. For each experiment, 2 mL (4%, vol/wt) of tempering solutions described above, including the water control, was added to 50 g of wheat. The centrifuge bottles were rotated continuously in a custom rotating mixer at ~24 rpm for 30 min and stored at ambient temperature for the remaining 17.5 h.

### Bacterial enumeration

The collected samples for the desiccation survival study were diluted in Butterfield’s buffer and homogenized by stomaching (Seward 400, Worthing, West Sussex, UK) for 1 min. Appropriate serial dilutions were performed using Butterfield’s dilution buffer, and the bacterial population was quantified using LBr agar (80 µg/mL). For the tempering experiment, the samples were diluted using D/E neutralizing broth (Neogen) to neutralize the disinfectant effect from the tempering solution prior to quantification.

### Modeling

The log-linear model (Equation 1) was first used to estimate the *D*-value of bacterial survival during storage. After we observed a decrease in the rate of pathogen reduction on wheat inoculated with cultures grown in broth (Fig. S2), the Weibull-Mafart’s model (45) (Equation 2) was used to address the tailing effect. The use of the Weibull model to describe desiccation survival of *Salmonella* and STEC in flour has been done previously (11, 12). Both models were estimated using ordinary least square (OLS) method.


(1) $$\log\left(\frac{N}{N_{0}}\right)=-\left(\frac{t}{D}\right)$$

(2) $$\log\left(\frac{N}{N_{0}}\right)=-{\left(\frac{t}{\delta }\right)}^{p}$$

In the log-linear model, two parameters are estimated using the collected data: the initial bacterial population log (*N*_0_), and the *D*-value, which is defined as the time (day) required to reduce bacterial population by 90% at a specific temperature, in this case, the storage temperature.

The parameter *P* was also estimated by the Weibull model, which represents the shape of the fitted curve; when *P* = 1, the curve is linear, whereas an increasing inactivation rate is reflected in *P* > 1, and *P* < 1 reflects a decreasing inactivation rate. The δ (day) represents the time for the first decimal reduction. To determine which model is more efficient for a given data set, the corrected Akaike information criterion (AIC_c_) (46) was calculated as:


(3) $$AIC_{c}=n\ln\left(\frac{SS}{n}\right)+2K+\frac{2K\left(K+1\right)}{n-K-1}$$

where *n* is the total number of data used for the estimation, SS is the sum of squares of residuals, and *K* is the number of parameters to be estimated in each model. The root mean squared error (RMSE) for each model was also calculated to assess the model fit. The parameters of the model were estimated using MATLAB (R2022a, The MathWorks Inc., Natick, MA). Both models were used to fit three biological replicates for each inoculum method and each bacterial strain after *a*_w_ equilibration. For each inoculum growth method/pathogen, 18 data points were used per biological replicate from day 2 to day 168. The initial log *N*_0_ for both models was also estimated, it was within 0.5 log CFU/g from the measured starting population, and therefore, it was omitted in Table 1. The estimated parameters were presented in Table 1 as average and standard deviation of three biological replicates to assess the variability for each treatment, while the RMSE and AICc were calculated by combining all data points from each biological replicate

### Statistical analysis

Prior to further analysis, the normality assumption was checked with the Shapiro-Wilk test using R (version 4.3.1). Depending on the number of predictor variables, the results were analyzed using the non-parametric Kruskal-Wallis test for overall significance within groups; in addition, Dunn’s test was used for pairwise multiple comparisons within group as a post hoc procedure following the rejection of the Kruskal-Wallis test. For larger data sets with multiple variables, the data sets were first normalized by transformation, and then the R package *rpart* (47) was used to build classification decision tree based on ANOVA tree. Due to the complexity of the data structure, two-way ANOVA was used to assist with further analysis. The tree was built based on normalized data; however, the mean value indicated in each leaf was transformed back manually for clarity.

## SUPPLEMENTAL MATERIAL

Supplemental material aem.00177-25-s0001.docx Table S1; Figures S1 to S3.

ASM does not own the copyrights to Supplemental Material that may be linked to, or accessed through, an article. The authors have granted ASM a non-exclusive, world-wide license to publish the Supplemental Material files. Please contact the corresponding author directly for reuse.
