# Supplementary material for: Inoculum growth impacts Salmonella and Shiga-toxin producing Escherichia coli resilience on wheat grain
Source: Appl Environ Microbiol. 2025 Mar 31;91(4):e00177-25. doi: 10.1128/aem.00177-25 (PMC12016515; doi:10.1128/aem.00177-25)
Supplement: Supplemental material — Table S1; Figures S1 to S3. [file aem.00177-25-s0001.docx]

Table S1. Average changes in bacterial population from inoculum to bacterial population on wheat grain after water activity equilibration. In each row, lowercase letters indicate significant (P < 0.05) difference among strains. In each column, capital letters indicate significant difference (P < 0.05) among inoculum growth methods

|  | *S.* PT30 | *S.* Typhimurium | *S.* Mbadanka | STEC O157:NM | STEC O26:H11 | STEC O121:H19 |
| --- | --- | --- | --- | --- | --- | --- |
| Broth | -2.0 ± 0.3 A a | -1.9 ± 0.2 AC a | -2.8 ± 0.2 A a | -2.3±0.3 AC a | -2.4±0.6 A a | -2.0±0.4 A a |
| Lawn-aerobic | -0.7 ± 0.5 B a | -0.5 ± 0.4 B a | -1.0 ± 0.5 B a | -0.8­±0.3 B a | -0.3±0.2 B a | -0.8±0.2 B a |
| Lawn-anaerobic | -0.7 ± 0.1 B a | -1.2 ± 0.2 AB a | -0.8 ± 0.7 B a | -0.8±0.1 B a | -0.9±0.1 B a | -1.1±0.1 B a |
| Acid-adapted | -2.6 ± 0.1 A a | -2.7 ± 0.4 C a | -2.4 ± 0.3 A a | -2.8±0.8 A a | -2.1±0.8 AC a | -1.8±0.3 AC a |
| Low inoculum | -0.7­ ± 0.2 B a | -1.4 ± 0.3 A a | -1.6 ± 0.5 AB a | -1.6±0.1 BC a | -1.2±0.1 BC a | -1.7±0.5 BC a |

Figure S1. Storage (A) temperature, (B) relative humidity, and the (C) water activity of wheat grain over 168 days.

Figure S2. Survival curves of pathogens on wheat across different inoculum growth methods over 168 days. In each plot, each point represents the average pathogen population of two techinical replicate, each color represents the result of one biological replicate. Solid lines show the predictive curve of log-linear model, dash lines show the predictive curve of Weibull model.

Figure S3. Morphology of bacterial colonies of (A) *Salmonella*, (B) STEC on Congo Red Agar, incubated at 30°C for 48 h.
